# Overview of European forensic youth care: towards an integrative mission for prevention and intervention strategies for juvenile offenders

**DOI:** 10.1186/s13034-019-0265-4

**Published:** 2019-01-14

**Authors:** Fleur Souverein, Tycho Dekkers, Elena Bulanovaite, Theo Doreleijers, Heidi Hales, Riittakerttu Kaltiala-Heino, Aurelio Oddo, Arne Popma, Nora Raschle, Klaus Schmeck, Marco Zanoli, Thimo van der Pol

**Affiliations:** 1 Department of Child- and Adolescent Psychiatry, Amsterdam University Medical Center (AUmc) Amsterdam, Amsterdam, The Netherlands; 2 Academic Workplace Forensic for at Risk Youth (AWRJ), Amsterdam, The Netherlands; 3 Department of Psychology, University of Amsterdam, Amsterdam, The Netherlands; 4 Department of Forensic Psychiatry and Complex Behavioral Disorders, De Bascule, Academic Center for Child- and Adolescent Psychiatry, Amsterdam, The Netherlands; 5 Department of Psychiatry, Child and Adolescent Psychiatry Sector, Hospital of Lithuanian University of Health Sciences, Kaunas, Lithuania; 6 Wells Adolescent Forensic Mental Health Unit, West London Mental Health Trust, London, UK; 7 Tampere University Hospital and Vanha Vaasa Hospital, University of Tampere, Tampere, Finland; 8 Ministry of Justice, Prison of Udine, Udine, Italy; 9 Department of Criminology, Leiden University, Leiden, The Netherlands; 10 Department of Child and Adolescent Psychiatry, Psychiatric University Hospital, University of Basel, Basel, Switzerland; 11 E.D.S.E.G., La Città dei Ragazzi, Modena, Italy; 12 Department of Forensic Youth Psychiatry, LUMC/Curium, Oegstgeest, The Netherlands; 13 Arkin, Amsterdam, The Netherlands

## Abstract

All over Europe youth delinquency is decreasing; our understanding of the factors related to juvenile delinquency and the characteristics of effective forensic youth care has increased substantially. However, effective prevention and intervention strategies are not always employed due to financial, demographical and socio-political challenges countries face, while the burden of mental health in juvenile justice populations is high. With this commentary, we highlight the importance of international collaboration to set out a direction to improve forensic youth care, to bundle our strengths and overcome our challenges. It is a continuation of the course that was set out by Doreleijers and Fegert (Child Adolesc Psychiatry Ment Health 5:20, 2011), in their editorial they highlighted the importance of collaboration and presented an overview of the state of the art on forensic youth care in eight European countries (and Russia). With this manuscript, we present an overview of statistics in juvenile justice of all European countries and present an integrated mission statement for forensic youth care, which was formulated in a keynote debate at the 6^th^ biennial congress of the European Association for Forensic Child and Adolescent Psychiatry, Psychology and other involved professions (EFCAP).

## Introduction

Across Europe (and in other Western countries) youth delinquency is decreasing [2]; due to a lack of referrals, the amount of juvenile correctional facilities has declined (e.g. for the Netherlands see: [19]. Scientific discoveries within the fields of criminology, sociology, psychology, pedagogics, psychiatry and neurobiology have given us a considerable amount of knowledge; our understanding of the factors related to juvenile delinquency and the characteristics of effective forensic youth care has increased substantially. There has been a general shift from ‘nothing works’ [16] to ‘what works’ (e.g. [15]) and ‘what works for whom’ (e.g. [3]).

Despite these positive developments, there is ample reason for concern. Youth within the juvenile justice system are among the most vulnerable citizens. The mental health needs in juvenile justice populations is high (e.g. [9]). Effective prevention and intervention strategies are not always employed due to financial, demographical and socio-political challenges countries face. Moreover, while the United Nations Convention of the Rights of the Child (UNCRC)—established in 1989 to protect the basic rights and special needs of youth—is the most ratified treaty worldwide (all countries except the United States), it is lamentably also the one most violated [24, 25]. Youth within the juvenile justice system continue to experience routine violations of their basic rights, including violence and isolation within detention centers [25]. The UNCRC provides an overarching legal framework and moral obligation to tackle these challenges, but within this framework a concrete and widely supported strategy (i.e. mission statement) for juvenile justice still needs to be set out.

The growing globalization offers opportunities for a global mission, or at least an integrated European mission, for juvenile justice. Researchers have remarked upon increasing policy transfers and a growing similarity in (juvenile) justice across western societies [20]. It is becoming more and more common for nations’ policymakers, practitioners and scientists to look worldwide to discover ‘what works for whom’ regarding forensic youth care. Notwithstanding these developments, the value of looking abroad for good policies and practices is still underestimated. With this commentary, we would like to highlight the importance of international collaboration to set out a direction to improve forensic youth care, to bundle our strengths and overcome our challenges. We hereby continue the course that was set out by Doreleijers and Fegert ([7], pg. 4), who stated that: ‘Especially in the field of forensic child and adolescent psychiatry, which is very much influenced by legal regulations in different countries, we think that an interdisciplinary international exchange is very important to improve care and rehabilitation of these youth’.

### Towards an integrative mission for Europa: a keynote debate at the 6th EFCAP congress

In June 2018, at the biennial congress of the European Association for Forensic Child and Adolescent Psychiatry, Psychology and other involved professions (EFCAP) in Venice, European countries united to formulate an integrated mission statement for forensic youth care. In a 2-h debate, keynote speakers from five European countries (Italy, England, Finland, Switzerland, and the Netherlands) each summarized the state of the art prevention and intervention strategies for juvenile offenders in their country and highlighted specific challenges accordingly. Each pitch was ended with an individual mission statement, followed by a debate in which the keynote speakers were challenged by the audience and debated with each other on how they wished to achieve the proposed missions. The debate was judged by a panel of young researchers and practitioners from different European countries (Italy, Switzerland, Lithuania and the Netherlands). At the end of the session, combining the information from the pitches and the debate, the panel presented an integrated mission statement for the future of forensic youth care in Europe.^1^ We consider the debate format that is presented in this paper to be an important and effective way of bringing different views of countries together and we strongly believe that the concluding mission statements are applicable to more than just these five countries. We consider this commentary to be a starting point for further European collaboration. In future endeavors, linked to the next biennial EFCAP congress in 2020, the authors aim to present an extensive and detailed overview of juvenile justice in Europe and present an integrated mission statement that accounts for all European countries.

### The current commentary

The current commentary presents an overview of statistics on juvenile justice in Europe (part 1) and presents the individual mission statements from the keynote speakers of the five European countries that participated in the debate at the 6th EFCAP congress (part 2); ultimately leading to an integrative mission statement for Europe lined out in five dispositions (part 3).

## Juvenile delinquency in Europe: an overview

Table 1 gives an overview of the statistics on juvenile detention in Europe [8]. It includes the number juvenile offenders held in detention measured at a certain date in 2016, related to the amount of adults in detention and ratio per 100.000 inhabitants, and includes the age of criminal responsibility for each country in Europe.

**Table 1:** Prisoner statistics ([8] Eurostat) in European countries

Country	Prisoners	Adult prisoners	Juvenile prisoners	N of prisoners per 100.000 inhabitants	N of juvenile prisoners per 100.000 inhabitants	Age of criminal responsibility
Albania	6.031	5.972	59	208,97	9,60	14
Austria	8.619	8.503	116	99,18	7,68	14
Belgium^a^	10.994	10.870	14	97,84	0,61	n/a^b^
Bosnia and Herzegovina^a^	2.832	2.214	11	Unknown	Unknown	Unknown
Bulgaria	7.345	7.318	27	102,67	2,27	14
Croatia	3.108	3.063	45	74,16	6,03	14
Cyprus	586	571	15	69,08	8,88	14
Czech Republic	22.481	22.396	85	213,01	4,48	15
Denmark	3.408	3.392	16	59,71	1,37	15
England & Wales	83.604	82.969	635	143,78	5,14	10
Estonia	2.864	2.835	29	217,64	11,74	14
Finland	3.156	3.063	93	57,51	8,67	15
France	68.432	67.674	758	102,50	5,11	13
Germany	64.291	63.020	Unknown	78,24	Unknown	14
Greece	9.560	9.310	250	88,65	13,32	15
Hungary	17.658	17.351	307	179,62	17,89	14
Iceland	116	116	0	34,88	0,00	15
Ireland^a^	3.716	3.150	60	78,65	4,98	12
Italy	55.978	54.653	1.325	92,27	13,24	14
Kosovo	1.648	1.589	59	93,02	10,08	Unknown
Latvia	4.243	4.200	43	215,49	12,21	14
Liechtenstein	72	72	0	191,38	0,00	14
Lithuania	6.815	6.757	58	235,93	11,19	14
Luxembourg	724	717	7	125,64	1,21	16
Macedonia	Unknown	Unknown	Unknown	Unknown	Unknown	Unknown
Malta	553	537	16	127,30	21,20	14
Montenegro	1.123	1.119	4	180,48	2,88	14
Netherlands	10.601	10.180	421	62,44	12,32	12
Northern Ireland	1.407	1.407	0	75,70	0,00	10
Norway^a^	4.192	4.116	3	80,45	0,27	15
Poland	71.528	70.041	1.487	188,39	21,72	13
Portugal	13.917	13.588	329	134,58	18,26	16
Romania	27.455	27.048	407	138,94	10,93	16
Scotland	7.744	7.282	462	144,57	44,80	8/12^c^
Serbia	10.672	10.433	239	150,81	19,49	14
Slovakia	9.995	9.919	76	184,20	7,60	14
Slovenia	1.308	1.299	9	63,37	2,48	14
Spain	59.589	57.711	650	128,31	7,79	14
Sweden^a^	5.910	5.527	16	59,99	0,81	15
Switzerland	6.541	6.522	19	78,55	1,27	10
Turkey	200.727	198.325	2.402	254,92	10,50	12

^a^Countries for which some information of 2015 was used because 2016 was not available

^b^No criminal law applies for youth under the age of 18

^c^In 2018 new legislation was proposed that will raise the age of criminal responsibility from 8 to 12

## The EFCAP debate: mission statements from 5 European countries

### Italy

The Presidential Decree 448/88 in Italy has set a course for a rehabilitative juvenile justice system focused on the (educational) development of young people and aims to reduce the amount of juveniles in detention by implementing several strategies, like offering alternative measures [6]. Forensic youth care in Italy, however, is still struggling with various issues [17]. First, the public debate on the right of existence of minor courts is ongoing, as many think minor courts should disappear and juveniles should be handled within the adult court. Second, social services are currently understaffed, therefore preventive examinations are rarely applied. Third, the large majority of youth in juvenile justice institutions are not receiving any kind of psychotherapy. Fourth, there is a plethora of institutions in the field of youth care, while communication between these services is almost non-existent. To counteract these challenges, it is recommended that there is a reduction in time between the delinquent act and the reaction of the system, as faster action could prevent many escalations. Furthermore, it seems crucial to involve youth in the justice system and properly explain the system to them. Moreover, schools should play a large role in prevention, including offering youth offenders alternative perspectives in life. Finally, it should be noted that refugees held for administrative reasons and juvenile delinquents oftentimes share the same facilities, although their needs are completely different. This observation is in close relation to current challenges refugees face in Italy; many of them are unaccompanied minors. All youth should get the care and support they need.

### England

The youth justice and health system in the England offers a wide range of prevention and intervention strategies for juvenile offenders. There are different residential secure facilities varying in levels of intensity of security and care. Recently, there have been several exciting developments in the provision of forensic youth care across all three levels of public health: (1) population-based interventions to reduce population risk factors such as deprivation and social exclusion; (2) interventions for at risk young people with risk factors; and (3) for those detained in secure settings. For example, at a population level, free school meals are now offered for all children in primary school, to enhance nutrition and thereby concentration at school and to reduce social exclusion. At level two, for those at risk of offending or starting to offend, forensic child and adolescent mental health services (FCAMHS; [5]) are now being rolled out across the country to assess and suggest interventions and Youth Offending Teams (YOTs; [22]) work hard to offer support and avoid incarceration. Finally, at the third level, regarding intervention for those already in secure care, there has been a recommendation by a Government review to move towards having secure schools instead of secure training centers or young offender institutions [22]. There is an ongoing debate about whether better care should be offered  within the youth justice settings or young people should be diverted into welfare or hospital settings; the most heated area of debate is how to care for young people who are at risk of developing personality disorders and those who are a high risk to themselves. Furthermore, there have been recent governmental reviews considering how the management of and care for young people in the justice system should be different from that of adults [12, 22]. Despite these positive developments, the structure of the youth justice system remains complex and referrals within this system often seem arbitrary [22]. Moreover, the ages of criminal responsibility (10 years) remains one of the lowest in Europe and there is a relatively large number of youth justice placements compared to other European countries (see Table 1). In order to tackle these issues, the aim should be to continue to improve young person centred multiagency service development for young people who have or are at risk of having contact with the criminal justice system.

### Finland

Finland in one of the leading countries in the world with regards to equality (e.g. see GINI Index World Bank^2^). There is stable economic development and political stability with consensual governance. In the recent years, many positive developments are noted: rates on substance abuse, bullying, delinquency, crime, teenage pregnancy and abortion have all dropped [13, 21]. In Finland, the age of criminal responsibility is 15 and at this moment a very small number of youth under the age of 18 are imprisoned in youth justice facilities. That is, child healthcare and welfare institutions take care of young delinquents. However, over the last years there is a worrisome increase of 30–40% in referrals to adolescent psychiatric services, an increase in mental health related visits to primary care and an increase in the proportion of children and adolescents included in special pedagogical support [18]. Focusing on the decline in delinquency, preliminary findings on bullying and substance abuse suggest that they might be increasing among those with lowest socio-economic status [23]. To counteract these negative developments, investments in schools and vocational education are needed. School attendance is a key predictor of positive development in children and adolescents [4, 14]. Educational paths should be tailored to the individual’s needs; school should be a place for everyone. Investing in pedagogical support at schools is necessary, so children at risk can overcome their difficulties and find their place in school, work, pro-social peer groups, and society.

### Switzerland

The approach on juvenile delinquency in Switzerland is focused on the offender, not on the offence. Offenders are investigated on several domains, such as developmental stage, personality and psychosocial situation. The age of criminal responsibility (10 years) is amongst the youngest in Europe. However, the aim of the juvenile justice system is to reintegrate juvenile offenders in society, not to retaliate. The Swiss system has several strengths: institutions are generally well funded, interventions exist at all levels of intensity, the psychotherapeutic approach is widely available and there is no differentiation between civil and criminal justice placements. In order to continuously improve the system, the Swiss ministry of justice funds applied research in juvenile institutions. Based on the Swiss system, it is recommended that prevention and intervention programs start early, focus on measures, invest enough money in the system (this pays off in the long run), and do research to improve the system.

### The Netherlands

Forensic youth care in the Netherlands is of high quality. It entails a wide range of evidence-based prevention and intervention strategies [27], with research studying its efficacy often incorporated in these interventions. In a broader perspective, the social security system provides a (financial) safety net, preventing many adolescents for going into forensic pathways. Between 2010 and 2017, the capacity of juvenile institutions reduced from 1240 to 505 [19]. However, considering forensic youth care within a larger societal view, there are also reasons for concern. Currently, moral political leadership is lacking which often results in an exclusive society. For example, ethnic minority youth in the Netherlands report increased externalizing behavior, which is associated with perceived discrimination and living in unstable social environments [1]. For the future of forensic youth care, we should model the right moral attitude. This attitude should entail unconditional love and epistemic trust [10], to create a more open, caring and inclusive atmosphere. In order to reach this goal, to stand up in the heat of the political debate, professionals in forensic child- and adolescent care should show that their work pays off. Calculating and monitoring cost-effectiveness of prevention and intervention programs is crucial in this respect [11, 26]. We should further invest in easily accessible care by creating informal and voluntary settings, where children can get advice or support and if indicated, but only with their consent, may be referred to forensic health care institutions.

## An integrated mission statement for Europe

Several common themes emerged from these mission statements and the debate following the pitches; leading to an integrated mission statement which is lined out in the following dispositions:

1. Forensic youth care should be viewed within a broader socio-political perspective: a safe society should be a caring and inclusive society. A society that offers the opportunities and perspective for all youth to flourish and develop to their full potential, considering the population of justice involved youth is becoming increasingly (culturally) diverse. Moreover, we must consider that our juvenile justice systems are politicalized and ‘tough on crime’ rhetoric with regard to forensic youth care is a popular strategy for political parties in the current polarizing political climate.
2. Invest enough money and show that it pays off: a sufficient financial investment should be invested in forensic youth care and research to further expand our knowledge on prevention and intervention strategies and to continuously improve them. Furthermore, research should focus on the larger economic effects of these strategies. Policies for forensic youth care should be based on pragmatic strategies judged on their (cost-) effectiveness.
3. Collaborate on national and international level: cross-talk between professionals, scientists and politicians should be pivotal. Value having multiple perspectives at the same issue (triangulation) and instead of focusing on differences, focus on our communal goal of fostering rehabilitation of juvenile offenders to promote optimal development and prevent recidivism. This includes, like the approach of the current paper, to bring stakeholders together and foster an active exchange of views, to highlight their common ground and commit every individual stakeholder to an integrative mission for the improvement of forensic youth care worldwide.
4. Prevention is crucial: integrate prevention and intervention strategies in educational systems and ensure equal educational opportunities for all youth. Invest in programs that offer easily accessible and voluntary care, support or advice.
5. The involvement of youth and their parents/caregivers should be a general principle: youth and their parents/caregivers should be involved in all aspects of forensic youth care, from research to policy making and from intervention development to setting out the individual trajectory during treatment (co-ownership). Empower them: make them part of the solution, instead of just the problem.

## Footnotes

1 For a graphical summary of the debate, see https://osf.io/93dr8/.

2 http://www.dataworldbank.org/.
